# Effects of Endurance Running Training Associated With Photobiomodulation on 5-Km Performance and Muscle Soreness: A Randomized Placebo-Controlled Trial

**DOI:** 10.3389/fphys.2019.00211

**Published:** 2019-03-05

**Authors:** Cecília Segabinazi Peserico, Alessandro Moura Zagatto, Fabiana Andrade Machado

**Affiliations:** ^1^Department of Physical Education, State University of Maringá, Maringá, Brazil; ^2^Department of Physical Education, School of Sciences, São Paulo State University–UNESP, Bauru, Brazil; ^3^Post-graduate Program of Physiological Sciences, Department of Physiological Sciences, State University of Maringá, Maringá, Brazil

**Keywords:** LED therapy, exercise training, aerobic exercise, athletic performance, performance-enhancing effects

## Abstract

This study aimed to investigate the influence of endurance running training associated with PBM on endurance performance variables and muscle soreness in untrained men. Thirty untrained men were distributed randomly into a placebo (PLA) group and photobiomodulation group (PBMG) and they performed 8 weeks of running training. The PBMG had the PBM performed before all training sessions. The PBM was applied using LED equipment with 56 diodes of red light (660 nm) and 48 diodes of infrared light (850 nm). The application was performed in 5 points per leg, with a dose of 60 J at each point and a total energy delivered per leg of 300 J. Peak running velocity, time limit tests and 5-km performance were assessed pre and post-training; muscle soreness was evaluated before all training sessions. The V_peak_ increased and 5-km running time (t_5–km_) decreased (*P* < 0.001) in both groups. In addition, the magnitude based-inference analysis showed a *possibly positive* effect on V_peak_ and t_5–km_ and for PBMG compared to PLA group. Furthermore, there was a moderate ES of 0.82 on attenuation in muscle soreness in the third week of endurance running training. Therefore, although the magnitude-based inference analysis demonstrated a *possibly positive* effect on V_peak_ and t_5–km_ and for PBMG compared to PLA group and a moderate ES on attenuation in muscle soreness in the last weeks of endurance running training, no significant difference were found between PBMG and PLA interventions.

## Introduction

Various methods have been used to optimize muscle recovery after exercise to facilitate the adaptations resulting from the endurance training (Vanin et al., 2018). The use of light-emitting diodes (LED), as well as low-level lasers, which comprise a photobiomodulation (PBM) modality, has shown many positive effects in accelerating the recovery process after exercise (Leal Junior et al., 2015; Ferraresi et al., 2016a; Vanin et al., 2018). In addition, PBM can be considered an ergogenic aid that positively affects oxidative metabolism by improving mitochondrial function (Ferraresi et al., 2015, 2016a; Vanin et al., 2018).

Recent human studies that associated PBM and acute aerobic exercise showed positive effects on physiological variables and endurance performance in the experimental condition (e.g., PBM) compared to placebo (PLA) (Dellagrana et al., 2018; Lanferdini et al., 2018a; Mezzaroba et al., 2018). For example, Dellagrana et al. (2018) found that all PBM doses tested (15, 30, and 60 J per site) positively affected running economy, rate of perceived exertion (RPE), velocity at maximal oxygen uptake (vVO_2max_), peak running velocity (V_peak_), and total time to exhaustion in recreational runners. In addition, Mezzaroba et al. (2018) investigated physically active men and reported that PBM applied prior to running enhanced maximum and submaximal VO_2_, increased the peak velocity (V_peak_), and reduced heart rate (HR), and Rating of Perceived Exertion (RPE) during incremental tests.

Although a longitudinal biological effect of PBM has been suggested, few studies in humans have examined the influence of PBM on human performance biomarkers with respect to endurance training (Paolillo et al., 2011, 2013; Vieira et al., 2012; Miranda et al., 2018). Paolillo et al. (2011, 2013) demonstrated significant improvements in post-exercise recovery parameters such as heart rate (HR) and muscle power of the lower limb in the PBM group in runners treated with LED during training compared to a control group. Miranda et al. (2018) also associated PBM (applied before and/or after each training session) with running training and reported positive effects on time to exhaustion and VO_2_ in healthy volunteers compared to the PLA group. However, these studies did not evaluate endurance running performance parameters such as 5-km running performance and V_peak_.

Another positive effect of PBM is on delayed onset muscle soreness (DOMS); PBM attenuated the increase in DOMS after a strength exercise session (Antonialli et al., 2014) and after an aerobic time trial (Machado et al., 2017). However, only Ferraresi et al. (2016b) evaluated DOMS longitudinally during a strength training program with PBM applied after all sessions and found that DOMS scores were lower in the PBM condition than in the PLA group 24 h after training sessions. Nevertheless, the effect of PBM associated with endurance running training on muscle soreness evaluated by DOMS after running training is not known and must be investigated, since PBM being considered a recovery method during exercise and will likely be used during the training season as a chronic intervention.

In this study, we aimed to investigate the influence of endurance running training associated with PBM on endurance performance variables and muscle soreness in untrained men. We hypothesized that the group with PBM application would present improved running performance and attenuated muscle soreness compared to the PLA group.

## Materials and Methods

### Participants

The sample size was calculated from *a priori* analysis for a group by time interaction comparison (F test, Anova for repeated measures, within-between interaction) according to an effect size of 0.52 (obtained from a pilot study), power of 80% and significance level of 5%. We used the software Gpower 3.1 (Düsseldorf, Germany) for the calculation. The *priori* power analysis revealed a minimal sample of 10 participants per group (*n* = 20). Volunteers were excluded if they used regular pharmacological agents or nutritional supplements, were smokers, were diagnosed with diabetes, hypertension, or asthma, presented any cardiovascular disorder, presented a body mass index ≥ 30 kg ⋅ m^−2^, or were engaged in other regular systematic physical training. Furthermore, only the participants who completed at least 90% of the training sessions were included in the final evaluation (Buchheit et al., 2010). Thus, 30 young and untrained men (aged between 20 and 35 years) volunteered to participate in this study. Participants were considered untrained if they had not engaged in running training or any other regular and systematic exercise training. Table 1 shows the characteristics of the study participants (mean ± *SD*).

**Table 1:** Participant’s characteristics for both PLA group and PBMG at pre-training.

Variables	PLA (*n* = 15)	PBMG (*n* = 15)
Age (years)	27.3 ± 5.2	27.4 ± 3.7
Body mass (kg)	80.2 ± 10.3	79.2 ± 7.0
Height (m)	1.8 ± 0.1	1.8 ± 1.0
Body fat (%)	17.2 ± 5.4	17.7 ± 5.7
Body mass index (kg ⋅ m^−2^)	25.7 ± 2.9	25.3 ± 2.5

*No statistical differences.*

This study was carried out in accordance with the recommendations of Permanent Committee on ethics in research with human beings (COPEP). The protocol was approved by the Local Human Research Ethics Committee (#623.581/2014). All subjects gave written informed consent in accordance with the Declaration of Helsinki.

**FIGURE 1:**
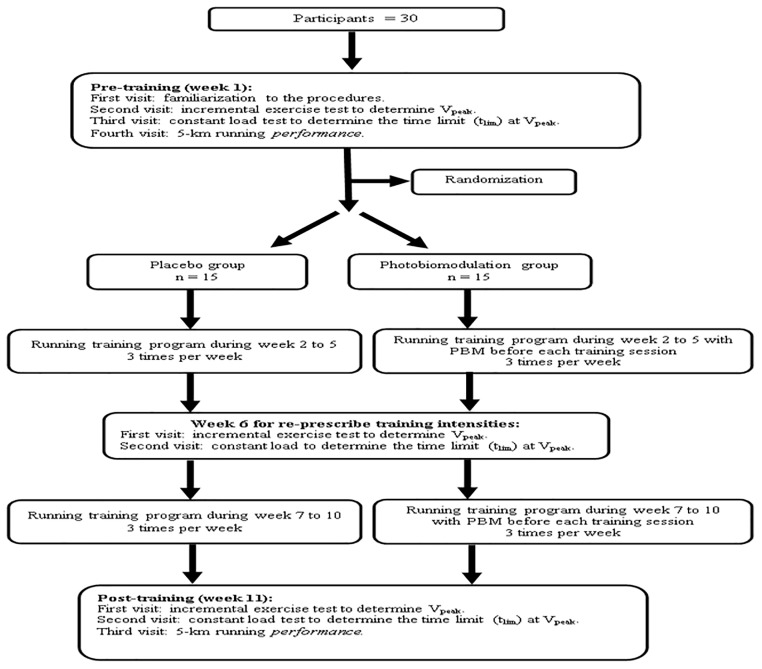
Flow chart of the study design.

The present study was a randomized double-blinded PLA -controlled experimental trial. The participants undertook four visits for baseline assessments (pre-training, week 1) where they underwent: (1) familiarization with the procedures, (2) incremental exercise tests to determine V_peak_, (3) a constant load test (rectangular test) to determine the time limit (t_lim_) at V_peak_, and (4) 5-km running performance. The first three visits were performed under laboratory conditions, and the tests were performed on a motorized treadmill (Super ATL; Inbrasport, Porto Alegre, Brazil). The 5-km running performance was performed on an outdoor track. All evaluations were performed within a maximum period of 7 days during which the participants did not perform training sessions. For the three maximal exercise tests, there was an intervening period of at least 48 h between tests to ensure the recovery of the participants between procedures. After the baseline assessments, the participants were randomly distributed in two experimental groups: the PLA group or the PBM group (PBMG). Both PLA and PBM groups performed the same running training program consisting of 8 weeks of endurance training, in which the only difference between the groups was that the PBMG received LED application before each training session. The V_peak_ test and its t_lim_ evaluation were repeated after 4 weeks of running training (i.e., from week 2 to 5) at week 6 to adjust training intensities during weeks 7–10 of training (i.e., the intensity prescription was based on V_peak_ and its t_lim_). During week 6 participants did not perform training sessions. Furthermore, all the assessments were repeated in the week after the end of the running training program (at post-training, week 11). Thus, the participants completed 11 weeks of training that consisted of 8 weeks of endurance running training and the other 3 weeks for assessment (week 1, 6, and 11). Figure 1 brings the study design.

### Incremental Exercise Test to Determine Peak Running Velocity (V_peak_)

After a warm-up, comprised walking at 6 km ⋅ h^−1^ for 3 min, the incremental protocol started with a velocity of 8 km ⋅ h^−1^ and increased by 1 km ⋅ h^−1^ between each successive 3-min stage until participants reached volitional exhaustion, with the gradient set at 1% (Machado et al., 2013; Peserico et al., 2015). This protocol was chosen because we previously demonstrated that this incremental rate and stage duration presented the highest correlations with endurance running performance and has been suggested as a tool for endurance running training prescription (Machado et al., 2013; Peserico et al., 2015). The V_peak_ of the incremental test was calculated as the velocity of the last complete stage added to the completed fraction of the incomplete stage (Kuipers et al., 2003), calculated according to the equation V_peak_ = V_complete_ + t/T × inc in which V_complete_ is the running velocity of the last complete stage, t the time in seconds sustained during the incomplete stage, T the time in seconds required to complete a stage, and inc is the speed increment. During the test, the HR (Polar RS800sd; Polar, Finland) and rating of perceived exertion (RPE) (Borg, 1982) were monitored and the maximal HR (HR_max_) and maximal RPE (RPE_max_) were defined as the highest HR and RPE values, respectively, obtained during the test.

### Constant Load Test to Determine the Time Limit (t_lim_) at V_peak_

After a 15-min warm-up at 60% of V_peak_, the treadmill velocity was quickly increased (within approximately 6 s) to the individual V_peak_ (Billat et al., 1996) and the treadmill gradient was set at 1%. Participants were encouraged to invest maximal effort and the time of permanency in this intensity was considered the t_lim_ at V_peak_. During the test HR and RPE were monitored and the HR_max_ and RPE_max_ were defined as the highest HR and RPE values, respectively, obtained during the test.

### 5-Km Running Performance

The 5-km time trial running performance was performed on a 400 m outdoor track and preceded by a self-determined warm-up of 10 min. Participants freely choose their pacing strategy during the performance. All participants were encouraged to give their best performance. The 5-km time for each participant were recorded and registered by the evaluator to determine the test duration (t_5–km_), and this result was considered the running performance of the participant.

### Muscle Soreness

For evaluated the perception of soreness we used a visual analog scale (VAS), which consisted of a 10 cm line labeled with “no soreness” on the left and “extremely sore” on the right. Previous studies have used a similar scale as a valid measure of muscle soreness (Baroni et al., 2010; Ferraresi et al., 2016a; Machado et al., 2017). This assessment was performed immediately pre-session (before the warm-up) in all training sessions. The pre-training session values of muscle soreness were considered DOMS from the preceding training sessions. The muscle soreness was elicited through a voluntary isometric contraction at 0 degrees of knee flexion and without load (Ferraresi et al., 2016b). Participants rated their perceived soreness related to the muscles involved in the isometric contraction (e.g., quadriceps, biceps femoris, and gastrocnemius) by placing a mark on the line that best corresponded to their perceived soreness and this assessment was quantified by measuring the distance in centimeters from the line on the left to the mark made by the participant.

### Endurance Running Training Program

Both groups performed all training sessions on a 400 m outdoor track during the afternoon and evening due to the availability of the participants on Mondays, Wednesdays, and Fridays, which meant that the participants performed training sessions three times per week. If for any reason they missed a training session, they re-scheduled for another weekday (usually Tuesday or Thursday) in order to perform at least 90% of the training (22 of the 24 training sessions prescribed). Participants were recommended to keep the same time of day for their training and testing as strictly as possible to avoid circadian cycle influence.

Training sessions consisted of moderate-intensity continuous training (MICT) and high-intensity interval training (HIIT). MICT and HIIT were both performed in the first (weeks 2–5) and last (weeks 7–10) training weeks. The MICT and HIIT were prescribed based on of V_peak_ and t_lim_ at V_peak_ determined during pre-training (week 1) and the exercise intensity was readjusted at week 6 (Table 2; Manoel et al., 2017). Training sessions were preceded by a 15 min warm-up, with 5 min of low self-selected intensity running, 5 min of stretching exercises, and 5 min of running at 60% of V_peak_. After each session, participants had 10–15 min of cool-down. In total, both groups of participants performed 24 training sessions on non-consecutive days over a period of 8 weeks (weeks 2–5 and weeks 7–10). They completed 8 weeks of training with MICT and HIIT training every other day. All training sessions were monitored by session-RPE and training load was quantified by multiplying the whole RPE using the 10-point scale (CR-10) by its duration (Foster, 1998).

**Table 2:** MICT and HIIT used during training sessions for both PLA group and PBMG.

**1st 4 weeks of training**	
MICT	30 ± 2.5 min at 75 ± 4% of V_peak_
HIIT	*X*^a^ series at 100 ± 2% of V_peak_ with duration of 60% of t_lim_ and intervals de 60% do t_lim_
**2nd 4 weeks of training**	
MICT	40 ± 2.5 min at 75 ± 4% of V_peak_
HIIT	*X*^a^ series a 100 ± 2% of V_peak_ with duration of 60% of t_lim_ and intervals of 60% do t_lim_

*^a^The number of series of each participant was adjusted for a duration of 30 ± 2.5 min (in the 1st 4 weeks of training) and 40 ± 2.5 min (in the 2nd 4 weeks of training).*

### Photobiomodulation (PBM)

Photobiomodulation was performed by LED application with a double-blind control, in which neither the participant nor the principal researcher knew about who received LED application or not. Thus, during the application the participants from both groups remained standing wearing a headset with music and blindfolded to avoid identification of the experimental group by audible and visual signals from the LED device. A second researcher controlled the groups, turning on the LED equipment (PBMG) or not (Placebo). The LED application was performed immediately before all training sessions (Vanin et al., 2016) and had a total duration of two and a half minutes (30 s per point, with application in both legs simultaneously), respecting the absence or presence of light emission for each group. For LED application, the method used was direct contact of the equipment with the site to be irradiated at an angle of 90° to the skin surface. This method has been previously used in other studies (Baroni et al., 2010; De Marchi et al., 2012).

The LED application was done on two regions of the quadriceps muscle, two regions of the biceps femoris, and one region of the gastrocnemius muscle, along the axis of muscle fibers distribution in both legs, and for 30 s each application point (De Marchi et al., 2012; Alves et al., 2014). LED was applied using an LED multidiode with cluster probe (THOR^®^ DD2 control unit, THOR, London, United Kingdom) with 56 diodes of red light (660 nm) and 48 diodes of infrared light (850 nm). The LED device information and application parameters are presented in Table 3.

**Table 3:** Parameters of photobiomodulation.

Number of LED diodes: 104 (56 red diodes; 48 infrared diodes)
Wavelength: Mixed, 660 nm (red diodes) and 850 nm (infrared diodes)
Frequency: Continuous; 0–1500 Hz
Optical output (for each diode): 10 mW (660 nm) and 30 mW (850 nm)
LED spot size (each diode): 0.2 cm^2^
LED cluster size: 46.3 cm^2^
Power density (for each diode): 50 mW/cm^2^ (660 nm) and 150 mW/cm^2^ (850 nm)
Energy density (for each diode): 1.5 J/cm^2^ (660 nm) and 4.5 J/cm^2^ (850 nm)
Application time: 30 s at each point
Energy: 60 J at each application point (0.3 J from each 660 nm diode; 0.9 J from each 850 nm diode)
Number of irradiation points per leg: 5
Total energy delivered per leg: 300 J

### Statistical Analysis

Data are presented as means ± standard deviations (SD) and were analyzed using the Statistical Package for the Social Sciences 17.0 software (SPSS Inc., United States). Initially, the Shapiro-Wilk test was used to check the normality of the data distribution. The variables were analyzed using mixed ANOVA for repeated measures. In addition, the Mauchly’s test of sphericity was applied and the Greenhouse-Geisser Epsilon correction was used when the sphericity criteria was not met. The analyses were completed with the Bonferroni *post hoc*. The main effects of group and the time in which the measurements were done and their interactions were also analyzed. The comparisons between groups at pre-training were made using the Student’s t test for independent samples. Statistical significance was set at *P* < 0.05.

In addition to conventional statistical analysis, magnitude-based inference analysis was used for comparisons between groups (Batterham and Hopkins, 2006) using spreadsheet designed for sports science research^1^. The values are expressed as the standardized mean difference (Cohen’s *d* ± confidence limits of 95%) (Cohen, 1988), which was calculated using pooled standard deviation as the denominator. The threshold values for ES were: <0.20 (trivial), 0.20–0.59 (small), 0.60–1.20 (moderate), >1.20 (large) (Hopkins et al., 2009). If the probabilities of the effect being substantially positive and negative were both >5%, the effect was reported as unclear, or, if not, the effect was clear. Thus, the changes were evaluated as follows: ≤1% most unlikely, >1–5% very unlikely, >5–25% unlikely, >25–75% possibly, >75–95% likely, >95–99% very likely, >99% most likely (Hopkins et al., 2009).

## Results

A total of 30 participants completed the study and there were no differences between groups at pre-training for all variables evaluated (*P* > 0.05). In addition, the mean training load was not different between groups for all MICT sessions[PLA = 304.7 ± 94.1 arbitrary unit (AU); PBMG = 317.9 ± 94.4 AU; *P* = 0.705] and for all HIIT sessions (PLA = 381.0 ± 94.7 AU; PBMG = 373.7 ± 93.7 AU; *P* = 0.834). In addition, the mean training loads calculated for each training week were not different between groups (*P* > 0.05).

**FIGURE 2:**
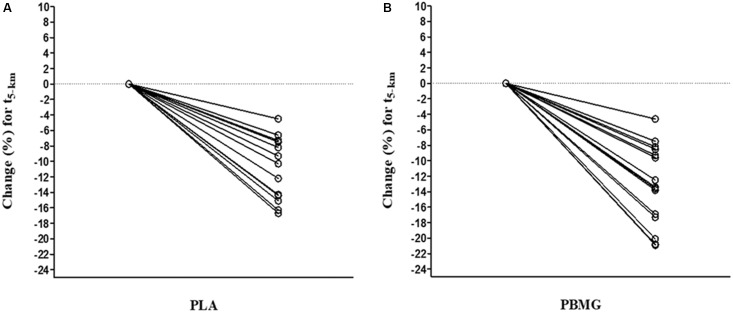
Individual change of time to complete 5 km (% change of t_5–km_) in PLA group and PBMG. **(A)** Individual change in the PLA group. **(B)** Individual change in the PBM group (*n* = 15 each group).

The results from pre- and post-training for the variables obtained during the incremental test, constant load test, and 5-km running performance are presented in Table 4. The mixed ANOVA for repeated measures revealed a significant main effect of time on the V_peak_, the HR_max_ from V_peak_ test, and from the t_lim_ test and t_5–km_; however, a group effect and group-time interaction was not found for all variables (*P* > 0.05). The V_peak_ increased in both groups (*P* < 0.001) with a moderate ES for the comparison between pre and post-training (PLA = 0.82 and PBMG = 1.01), whereas the HR_max_ obtained in the incremental test decreased in both groups (*P* < 0.001). The t_lim_ did not change post-training for PLA and PBMG; however, the HR_max_ from the t_lim_ test decreased in the PBMG (*P <* 0.001). The 5-km running time (t_5–km_) decreased in the PLA group and PBMG (*P* < 0.001) with a moderate ES for the comparison between pre and post-training (PLA = −0.82 and PBMG = −1.18) The magnitude based-inference analysis showed a *possibly positive* effect on V_peak_ and t_5–km_ and for PBMG compared to PLA group (Table 4). In addition, to illustrate a greater result in the PBMG for the t_5–km_, Figure 2 brings individual change of t_5–km_ from each group.

**Table 4:** Performance variables obtained from the V_peak_ test, t_lim_ test, and 5-km running performance for both groups at pre and post-training.

Variables	PLA (*n* = 15)			PBMG (*n* = 15)					Magnitude-based inference analysis		
	Pre-training	Post-training	Cohen’s ES pre × post [CI 95%]	Pre-training	Post-training	Cohen’s ES pre × post [CI 95%]	Time effect (F; *P*)	Interaction Time × group (F; *P*)	Cohen’s ES [CI 95%]	% chances (positive/trivial/negative)	Qualitative inference
V_peak_ (km ⋅ h^−1^)	13.4 ± 1.1	14.4 ± 1.0^∗^	0.82 [0.65 – 1.00]	13.4 ± 1.2	14.6 ± 1.0^∗^	1.01 [0.77 – 1.25]	178.39;<0.001	2.282; 0.142	0.21 [−0.08 – 0.50]	53/47/0	Possibly positive for PBMG
HR_max_ from V_peak_ test (bpm)	193 ± 9.3	187 ± 7.3^∗^	−0.56 [−0.87 – −0.26]	196 ± 10.7	189 ± 9.8^∗^	−0.56 [−0.68 – −0.44]	59.733; < 0.001	0.271; 0.606	−0.08 [−0.38 – 0.23]	4/76/20	Very Unlikely
RPE_max_ from V_peak_ test (6–20)	19.7 ± 0.6	19.9 ± 0.4	0.31 [−0.17 – 0.38]	19.7 ± 0.8	19.9 ± 0.3	0.31 [−0.20 – 0.82]	3.430; 0.075	0.070; 0.793	0.09 [−0.60 – 0.78]	37/43/20	Unclear
t_lim_ (min)	6.6 ± 0.7	6.9 ± 1.1	0.46 [−0.62 – 1.53]	6.6 ± 1.1	6.8 ± 1.2	0.23 [−0.47 – 0.92]	1.290; 0.266	0.013; 0.910	−0.06 [−1.18 – 1.06]	32/28/40	Unclear
HR_max_ from t_lim_ test (bpm)	188 ± 9.6	186 ± 8.0	−0.18 [−.048 – 0.11]	192 ± 10.6	187 ± 9.5^∗^	−0.42 [−0.56 – −0.28]	17.983;<0.001	3.393; 0.076	−0.27 [−0.57 – 0.03]	0/32/67	Possibly positive
RPE_max_ form t_lim_ test (6–20)	19.9 ± 0.3	19.8 ± 0.4	−0.49 [−1.54 – 0.56]	19.9 ± 0.4	19.9 ± 0.5	0.00 [−0.59 – 0.59]	0.606; 0.443	0.606; 0.443	0.41 [−0.64 – 1.47]	66/22/12	Unclear
t_5–km_ (min)	27.0 ± 3.3	24.1 ± 2.5^∗^	−0.82 [−1.04 – −0.61]	27.6 ± 3.0	23.9 ± 2.2^∗^	−1.18 [−1.48 – −0.88]	135.46;<0.001	2.218; 0.148	−0.26 [−0.61 – 0.10]	1/37/63	Possibly positive for PBMG

*PLA, placebo group; PBMG, PBM group; ES, effect size; V_peak_, peak running velocity; HR_max_, maximal heart rate; RPE_max_, maximal rating of perceived exertion; t_lim_, time limit; t_5-km_, 5-km time. ^∗^P < 0.05 compared with pre-training in the same group.*

Table 5 brings the results from pre-session muscle soreness evaluated by VAS during 8 weeks of endurance running training (weeks 2–5 and weeks 7–10). It was not demonstrated a group effect, time effect and group-time interaction (*P* > 0.05) on the muscle soreness values. In addition, a moderate ES of 0.82 was found for the comparison between groups (1.2 ± 1.3 cm vs. PLA and 0.6 ± 0.6 cm for PBMG) in the third week of endurance running training (week 4). The magnitude based-inference analysis showed *unclear* effect on muscle soreness values in all training weeks, except for the third week that presented a qualitative inference of *very unlikely* (Table 5).

**Table 5:** Muscle soreness responses evaluated by visual analogic scale (VAS) during weeks of running training.

Training weeks	PLA (*n* = 15) (cm)	PBMG (*n* = 15) (cm)	Cohen’s ES [CI 95%]	% chances (positive/trivial/negative)	Qualitative inference
2	1.1 ± 1.4	0.9 ± 0.8	0.22 [−0.65 – 1.09]	52/32/16	Unclear
3	1.2 ± 1.1	1.0 ± 0.8	0.26 [−0.54 – 1.05]	56/32/12	Unclear
4	1.2 ± 1.3	0.6 ± 0.6	0.82 [−0.43 – 2.07]	85/10/5	Very Unlikely
5	0.8 ± 1.0	1.0 ± 0.8	−0.16 [−0.84 – 0.53]	14/41/45	Unclear
7	1.0 ± 0.9	1.2 ± 1.2	−0.15 [−0.77 – 0.47]	12/45/43	Unclear
8	0.8 ± 0.9	1.0 ± 1.0	−0.14 [−0.71 – 0.44]	11/48/41	Unclear
9	0.9 ± 0.7	0.8 ± 0.7	0.08 [−0.48 – 0.65]	33/51/15	Unclear
10	0.9 ± 1.0	0.7 ± 0.9	0.20 [−0.40 – 0.79]	50/42/9	Unclear

*PLA, placebo group; PBMG, PBM group. No statistical differences.*

## Discussion

The present study aimed to investigate the influence of endurance running training associated with PBM on endurance performance variables and muscle soreness in untrained men. The main finding was that although the magnitude-based inference analysis demonstrated a *possibly positive* effect on V_peak_ and t_5–km_ and for PBMG compared to PLA group and a moderate ES on attenuation in muscle soreness in the third week of endurance running training, no significant difference were found between PBMG and PLA interventions.

The data obtained from the incremental test demonstrated that V_peak_ and t_5–km_ improved in both groups (pre to post-training). Additionally, our findings highlighted that, despite the differences between groups not being statistically significant, PBM had a *possibly positive* effect on improving performance variables compared to PLA. Previous transversal studies reported better V_peak_ improvements with PBM conditions compared to that under the PLA conditions (Dellagrana et al., 2018; Mezzaroba et al., 2018). Another important physiological adaptation observed in our study was the decrease of the HR_max_ values obtained from the maximal tests in both groups; it was postulated that HR_max_ can be altered by 3–7% with an ES of –0.48 after aerobic training (Zavorsky, 2000).

Other human studies also found that PBM caused greater improvements in variables related to aerobic capacity after training than PLA or control group (Paolillo et al., 2011, 2013; Miranda et al., 2018), however, none of them investigated running performance on a time trial. Miranda et al. (2018) examined the effects of PBM applied at different moments (before and/or after each training session) and concluded that PBM applied before and after sessions can improve variables evaluated during progressive cardiopulmonary tests on a treadmill (e.g., VO_2_ and time to exhaustion) when compared to the PLA.

Studies by Paolillo et al. (2011, 2013) investigated the effects of PBM application in post-menopausal women during training sessions and found positive effects of PBM on muscle power evaluated by isokinetic testing (Paolillo et al., 2011), maximal performance during the Bruce protocol and fast post-exercise recovery assessed by the time in which the HR and blood pressure returned to baseline values (Paolillo et al., 2013).

In contrast, Vieira et al. (2012) examined the effects of a cycle ergometer training program with PBM applied after each training session on isokinetic variables in healthy women. The authors found that only the low-level laser group had a significant decrease in the fatigue index of the knee extensor muscles. However, it is important to note that these studies (Paolillo et al., 2011, 2013; Vieira et al., 2012) did not perform evaluations or tests whose results could be extrapolated to running performance or assess aerobic capacity; thus, our study is the first to present endurance running-related variables. Furthermore, using animal models, Guaraldo et al. (2016) investigated the effects of 6 weeks of swimming aerobic training in conjunction with low level laser application before all training sessions, and reported that the training group which received low level laser presented greater improvements in aerobic performance, represented by the VO_2max_ and V_peak_ variables, compared to the other control groups.

Based on our results and previous data that showed the positive effects of PBM application on aerobic parameters (Dellagrana et al., 2018; Lanferdini et al., 2018a; Miranda et al., 2018) we suggest that PBM can be an ergogenic aid with positive effects on oxidative metabolism. There are several possible physiological mechanisms to explain these positive effects (Leal Junior et al., 2015); for example, the increased blood flow and the increased activity of oxidative enzymes such as cytochrome c oxidase inside the mitochondria leading to increased ATP synthesis (Albuquerque-Pontes et al., 2015; Ferraresi et al., 2015).

Concerning the muscle soreness results (i.e., DOMS), although the comparisons did not demonstrate statistical differences between groups, a moderate ES of 0.82 was observed for the comparison between groups in the third week of endurance running training (Table 5). Previous studies also reported that PBM applied before maximal exercise of knee eccentric contractions (Antonialli et al., 2014) and after running time trials (Hausswirth et al., 2011; Machado et al., 2017) had a greater ability to reduce DOMS compared to PLA conditions. In contrast, using the low-level laser application before a maximal lower limb resistance exercise, Baroni et al. (2010) did not demonstrate significant differences between the DOMS responses in the PLA group and the low level laser group. Only Ferraresi et al. (2016a) evaluated DOMS during a 12-week strength training program by applying the visual analog scale 24 h after the first, 13th, 25th, and 36th training sessions; lower visual analog scale scores were reported by the participants with low level laser application after all sessions compared to those under PLA conditions.

Despite the important findings, the present study had some limitations; for example, the lack of another control group with only the PBM application and without endurance running training. Furthermore, it is important to note that because different PBM parameters (e.g., wavelength, time to apply PBM, dosage) influence the responses of different variables (Lanferdini et al., 2018b; Miranda et al., 2018), we suggest that the dosage and the time of application used could influence the magnitude of our results. For example, Ferraresi et al. (2016a) in a recent review on PBM in human muscle tissue, reported some evidence in favor of applying PBM before exercise in association with training programs, as used in our study, to increase performance, limit muscle damage and prevent pain from 1 h until 72–96 h after exercise. However, concerning the time that elapses between application of the PBM and the exercise performance, it was demonstrated that PBM applied 3–5 min before a bout of exercise may not actually be the best time point and that a longer duration before exercise may be more favorable (Ferraresi et al., 2016a). However, it is important to emphasize that this assumption is not completely clear in the literature and that further investigations could answer this question (Ferraresi et al., 2016a).

Therefore, we concluded that although the magnitude-based inference analysis demonstrated a *possibly positive* effect on V_peak_ and t_5–km_ and for PBMG compared to PLA group and a moderate ES on attenuation in muscle soreness in the third week of endurance running training, non-significant differences were found between the PBMG and PLA interventions.

## Practical Application

In terms of practical application, these results demonstrated that, despite being non-significant, there is the possibility for using PBM as strategy for practitioners and recreational runners during endurance running training to optimize their adaptations. In sports performance small improvements can determine the winner or loser. However, further research is required to examine the chronic effects of endurance running training with different PBM doses and analyze other biomarkers related to muscle soreness, inflammation, and oxidative metabolism.

## Author Contributions

CP and FM designed the work and acquired the data. CP, AZ, and FM contributed to the analysis and interpretation of the data. CP and FM drafted the work. AZ revised it critically. All authors approved the final version of the manuscript.

## Conflict of Interest Statement

The authors declare that the research was conducted in the absence of any commercial or financial relationships that could be construed as a potential conflict of interest.

## Footnotes

1 http://www.sportsci.org
